# c-di-AMP–DasR signaling axis mediates mycobacterial acid resistance

**DOI:** 10.1128/mbio.03708-25

**Published:** 2026-02-06

**Authors:** Yu Fu, Xue-Qin Xie, Zhan-Hui Xu, Yi-Fan Liang, Shi-Qi Yang, Bang-Ce Ye, Di You

**Affiliations:** 1 Laboratory of Biosystems and Microanalysis, State Key Laboratory of Bioreactor Engineering, East China University of Science and Technology 543684 , Shanghai, China; The University of Texas Health Science Center at Houston, Houston, Texas, USA

**Keywords:** c-di-AMP, c-di-AMP receptor, DasR

## Abstract

Pathogenic mycobacteria encounter acidic environments during host invasion, necessitating sophisticated acid resistance mechanisms. Here, we identify the GntR family regulator DasR as a conserved cyclic di-AMP (c-di-AMP) receptor in *Mycobacterium tuberculosis* that orchestrates acid adaptation through a multilayer network. Biochemical analyses demonstrated that DasR binds c-di-AMP with 20-fold higher affinity under acidic conditions than under neutral conditions, as evidenced by a Kd shift from 226 μM to 11.4 μM. This pH-sensitive binding aligns with acidified host niches during infection. ChIP-seq revealed that DasR directly targets nucleotide second-messenger metabolism genes, dynamically balancing intracellular pools of (p)ppGpp, cyclic AMP (cAMP), and c-di-AMP via positive feedback regulation. Concurrently, DasR upregulated the expression of the molecular chaperone HtpG, which stabilizes the DasR complex under acid stress. Functionally, c-di-AMP enhances DasR-DNA binding capacity at low pH, whereas HtpG-mediated thermostability amplifies signal output. This integrated axis coupling pH sensing, transcriptional reprogramming of stress metabolites, and chaperone reinforcement confers robust acid resistance. These findings establish the c-di-AMP–DasR pathway as an evolutionarily optimized strategy for mycobacterial persistence in hostile environments and suggest that this axis could be targeted to disrupt *M. tuberculosis* resilience.

**IMPORTANCE:**

The findings identified a regulatory axis central to mycobacterial acid adaptation, and DasR was found to be a conserved c-di-AMP receptor in *Mycobacterium tuberculosis*. A key feature is its highly pH-sensitive binding to c-di-AMP, which exhibits a 20-fold increase in affinity under acidic conditions, indicating that environmental cues are linked to the transcriptional response. DasR directly targets genes governing (p)ppGpp, cyclic AMP (cAMP), c-di-AMP metabolism, and acid adaptation, creating a feedback loop that dynamically balances stress signaling pathways. The concurrent upregulation of the chaperone HtpG stabilizes the DasR complex, increasing signal output under stress. This integrated system, which combines allosteric enhancement of DNA binding with chaperone-mediated stabilization, constitutes an evolutionarily refined strategy for acid resistance. The c-di-AMP–DasR pathway is therefore a promising target that could enable researchers to address the persistence of *M. tuberculosis*.

## INTRODUCTION

The intracellular pathogen *Mycobacterium tuberculosis* encounters extreme acidification (pH ~ 4.5) within host macrophage phagosomes—a lethal environment that disrupts bacterial membrane integrity, inhibits metabolic enzyme activity, and triggers protein denaturation (1). This acidic assault is a host defense mechanism that is conserved across vertebrates, in which the phagosomal pH decreases below 5.0 within 15–60 min of pathogen internalization (2, 3). Studies have demonstrated that *M. tuberculosis* mutants defective in acid resistance exhibit >100-fold reduced survival in macrophages, underscoring that pH adaptation is a nonredundant virulence trait (1, 4, 5). Although mechanisms such as urease-mediated neutralization or proton pump extrusion are characterized in other gram-positive bacteria, the strategy used by *M. tuberculosis* to sense and counteract acidic stress remains incompletely resolved, particularly with respect to the master regulators that integrate environmental cues with transcriptional reprogramming (6, 7).

Central to bacterial stress adaptation are nucleotide second messengers. Among these molecules, cyclic AMP (cAMP) has been extensively studied because of its critical regulatory functions in *M. tuberculosis*. Specifically, intracellular cAMP levels modulate gene expression in response to environmental cues such as hypoxia (8), and the deletion of Rv3676, a gene encoding a cAMP receptor protein (CRP) family transcription factor, alters virulence-associated gene expression *in vivo* (9). Concurrently, cyclic di-AMP (c-di-AMP) serves as a widespread second messenger that coordinates cell wall homeostasis, osmolyte transport, and antibiotic persistence across Actinobacteria. In mycobacteria, DisA-mediated c-di-AMP synthesis is essential for viability, with dysregulation affecting virulence and overall fitness (10). c-di-AMP interacts with intracellular receptors, including transcription regulators, effector proteins, and riboswitches. Elevated c-di-AMP levels in *M. tuberculosis* inhibit intracellular replication by inducing autophagy (11) and concurrently regulate essential processes, such as cell wall metabolism, ion homeostasis, and DNA damage repair (12). Furthermore, c-di-AMP serves as a crucial virulence determinant, with its concentration directly influencing bacterial survival and pathogenic efficacy (13). Both excessive and insufficient amounts of c-di-AMP disrupt mycobacterial membrane integrity, increasing susceptibility to osmotic lysis (14). Notably, high c-di-AMP concentrations attenuate bacterial virulence, highlighting the potential of this molecule as a therapeutic target for tuberculosis (15, 16).

Paradoxically, despite these pleiotropic functions, the role of c-di-AMP receptors in mediating pH adaptation in mycobacteria remains unidentified. Notably, although *Bacillus subtilis* employs KhtTU for c-di-AMP and pH-dependent potassium uptake (17), such a dedicated mechanism has not been identified in *M. tuberculosis*. Although DisA activity is suggested to be pH sensitive (18), the regulatory mechanism and physiological implications of this sensitivity remain unclear in mycobacteria, indicating a mechanistically unresolved connection between c-di-AMP signaling and acid stress resistance.

Here, we identify the GntR family transcriptional regulator DasR as a pH-sensing c-di-AMP receptor essential for acid resistance in *M. tuberculosis*. Electrophoretic mobility shift assay (EMSA) and isothermal titration calorimetry (ITC) revealed a 20-fold enhanced affinity of DasR for c-di-AMP under acidic stress, mirroring phagosomal conditions during infection. Genome-wide ChIP-seq demonstrated that ligand-bound DasR directly occupies the promoters of nucleotide second-messenger genes (*relA*, *cya*, *disA*, etc.), dynamically balancing (p)ppGpp, cAMP, and c-di-AMP pools while upregulating the expression of the chaperone *htpG*. This orchestrates a self-reinforcing circuit: c-di-AMP allosterically potentiates DasR-DNA binding more than eightfold, which is amplified at low pH; transcriptional activation of *disA* autoamplifies c-di-AMP synthesis; and HtpG stabilizes the receptor complex under acidic stress. Collectively, this triad of pH-sensing affinity shifts, stress-metabolite network rewiring, and chaperone-mediated complex stabilization constitutes an evolutionary innovation enabling mycobacterial persistence in hostile niches. Thus, targeting DasR-c-di-AMP interactions may be a promising antimicrobial strategy for tuberculosis.

## RESULTS

### DasR is a pH-sensitive c-di-AMP receptor in mycobacteria

**Fig 1:**
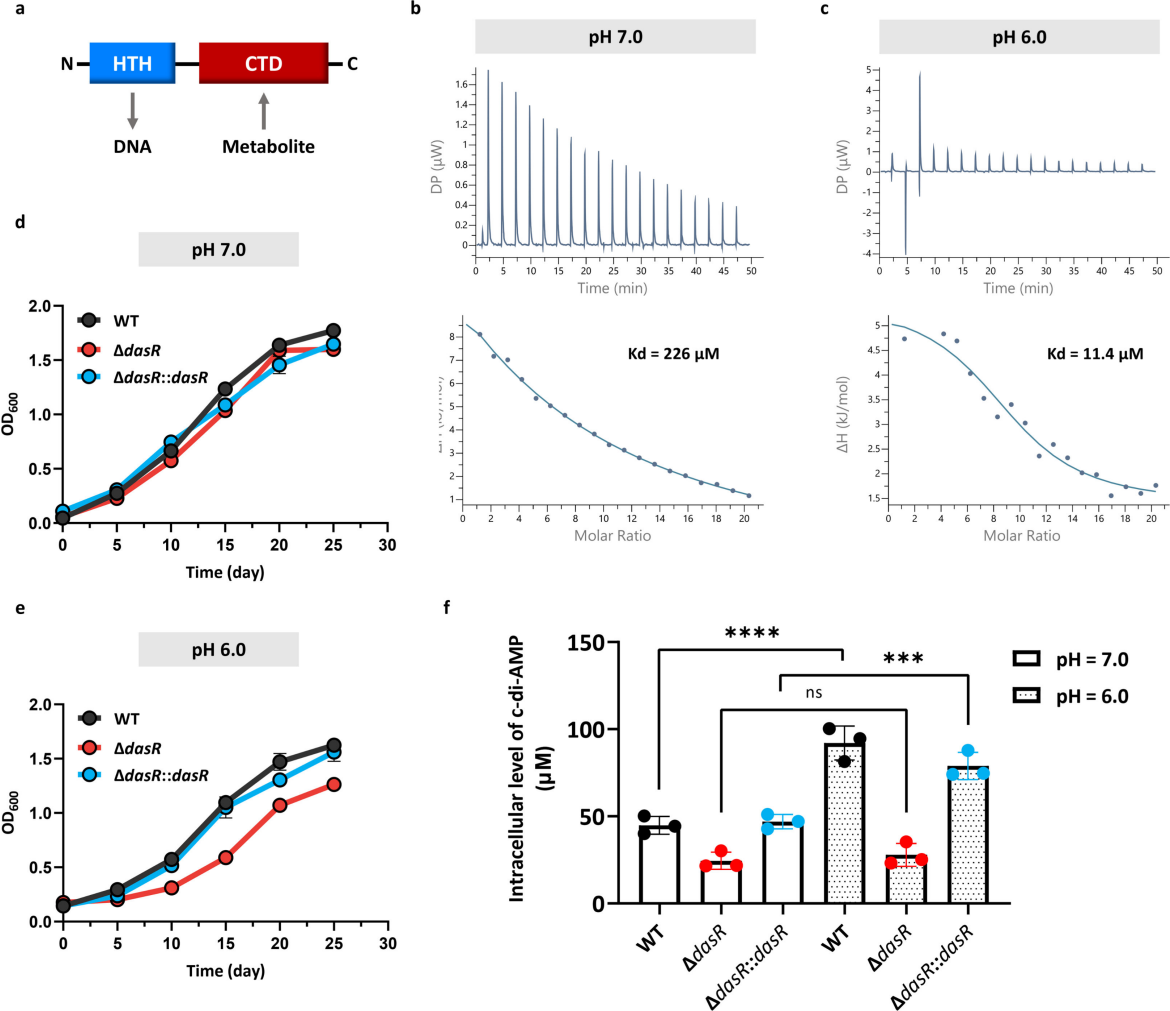
DasR is a pH-sensitive c-di-AMP receptor. (**a**) Schematic representation of GntR family protein domain organization showing the DNA-binding domain (HTH, blue) and effector-binding domain (CTD, red). Characterization of the interactions of c-di-AMP with DasR using ITC. Titration of nucleotides (1 mM) into DasR (10 μM). ITC measurements at pH 7.0 (**b**) and pH 6.0 (**c**). Growth curves of the *M. tuberculosis* H37Ra WT, Δ*dasR*, and dasR-complemented (Δ*dasR::dasR*) strains at pH 7.0 (**d**) and pH 6.0 (**e**). (**f**) Intracellular c-di-AMP concentrations in *M. tuberculosis* H37Ra strains grown in liquid MB7H9 medium during late exponential growth (OD_600_ = 1.0). The c-di-AMP concentrations of the samples were normalized to the dry cell weight. Data are presented as the means ± SDs for *n* = 3 biological replicates. ***P < 0.001; ordinary one-way analysis of variance was used for the statistical test.

Recent studies have reported DasR as a c-di-AMP receptor protein in *Streptomyces* and suggested its potential conservation across Actinobacteria (19). Comparative analysis revealed that its mycobacterial homolog shares 25% sequence identity with *Streptomyces* DasR and exhibits characteristic architectural features of the HutC subfamily within the GntR transcriptional regulator family. *M. tuberculosis* DasR is a canonical two-domain protein comprising (i) a smaller N-terminal domain with a conserved winged helix-turn-helix (HTH) motif for DNA binding and (ii) a larger C-terminal domain (CTD) that facilitates effector binding and mediates oligomerization (Fig. 1a). To test whether DasR is a potential c-di-AMP receptor protein, His-DasR with c-di-AMP, ADP, AMP, or cAMP was subjected to an ITC assay. As shown in Fig. 1b and c, the affinity of DasR for c-di-AMP was 20-fold greater under acidic conditions (Kd = 11.4 µM) compared with neutral conditions (Kd = 226 µM). In contrast, no binding signals were detected in response to ADP, AMP, or cAMP (Fig. S1).

Having established the specific binding, we next sought to characterize its structural basis and functional role. Molecular docking suggested that the CTD was the c-di-AMP binding site (Fig. S2), and we further characterized the binding interface and dimeric structure. We then evaluated the phenotypic consequences of *dasR* deletion and found that the Δ*dasR* mutant displayed a growth defect at low pH (Fig. 1d and e). Furthermore, this phenotype was linked to impaired c-di-AMP accumulation during acid stress (Fig. 1f), suggesting that DasR is required for bacterial survival under acidic conditions. Collectively, these results establish DasR as a c-di-AMP receptor protein in *M. tuberculosis* and suggest that the c-di-AMP–DasR signaling axis likely plays a central role in mycobacterial acid resistance.

### Genomic mapping of DasR binding sites unifies acid–stress adaptation pathways

**Fig 2:**
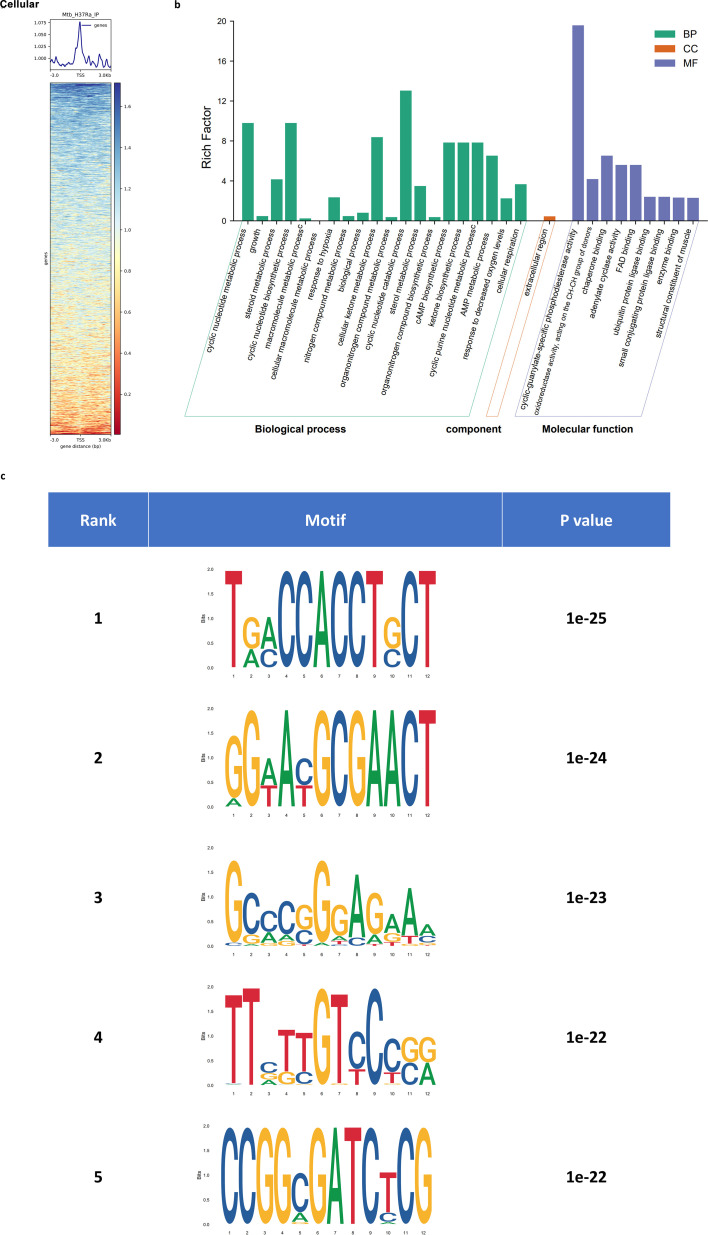
Genomic mapping of DasR binding sites unifies acid–stress adaptation pathways *in vivo*. (**a**) Heatmaps of the ChIP-seq signal density at the peak center and TSSs (±3 kb). The average signal profile is shown. Red indicates a low signal, and blue indicates a high signal. (**b**) GO terms and enrichment analysis for DasR target genes in the network. (**c**) Motif analysis shows the best-matched motifs occupied by DasR.

To confirm and extend the function of the c-di-AMP–DasR signaling axis to cells, we used chromatin immunoprecipitation sequencing (ChIP-seq) to determine DasR-dependent binding throughout the genome in *M. tuberculosis*. The wild-type *M. tuberculosis* H37Ra strain was grown in liquid MB7H9 medium and subjected to ChIP-seq experiments using a specific anti-DasR antibody (custom-produced by Beyotime Biotechnology, Shanghai, China). The total (nonimmunoprecipitated) input DNA was also subjected to sequencing. As shown in Fig. 2a, the signals were widely distributed at transcription start sites (TSSs), with a sharp single peak. GO analyses of the target genes revealed enrichment in nucleotide metabolism, nitrogen metabolism, and chaperone binding in diverse environments (Fig. 2b). In addition, motif analysis revealed that the **T**ga**CCACCT**g**CT** motif matched the top hit (*P* value 1e^−25^) at the DasR peak (Fig. 2c). These results implied that DasR directly targets genes governing nucleotide second-messenger dynamics, including the (p)ppGpp synthases/hydrolases RelA (ATP/GTP pyrophosphatase) (20) and Ppx1/2 (exopolyphosphatases), which collectively regulate the intracellular (p)ppGpp pool; the cAMP producers Cya (adenylate cyclase) and CpdA (cAMP phosphodiesterase), which are responsible for cAMP synthesis and degradation (21, 22); and the c-di-AMP synthase DisA (Fig. S3a through c). Moreover, ChIP-seq data revealed that DasR binds directly to the promoter regions of genes with established roles in acid adaptation, including *drrAB*, which encodes an ABC (ATP-binding cassette) transporter critical for intracellular pH homeostasis (23), and *ripA*, a peptidoglycan DL-endopeptidase important for the regulation of cell envelope composition and virulence under acidic stress (24, 25) (Fig. S3d). These findings position DasR as a master coordinator of bacterial stress signaling, capable of synchronizing multiple second-messenger pathways under acid stress.

### DasR orchestrates nucleotide second-messenger homeostasis through direct transcriptional control

**Fig 3:**
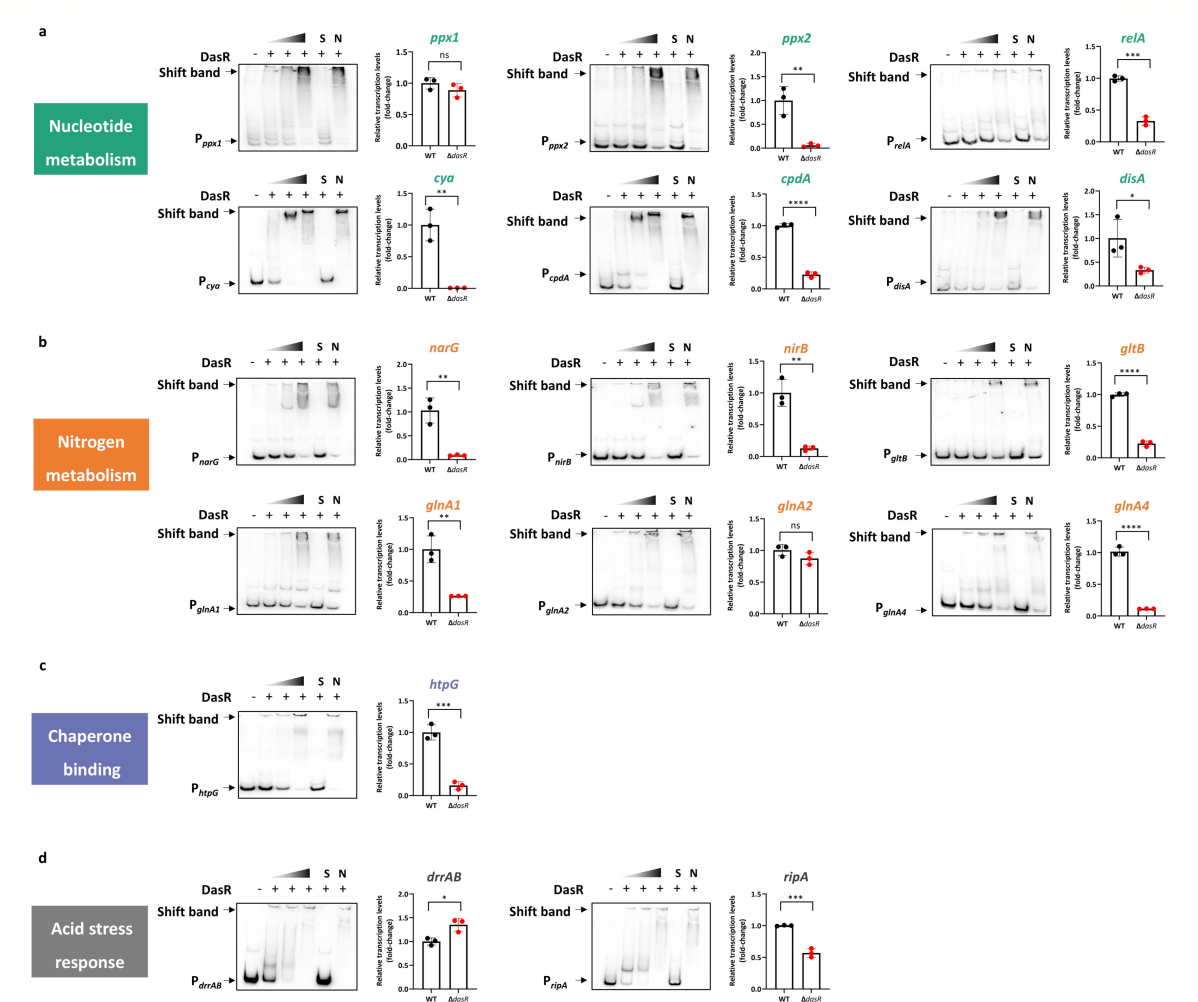
DasR directly activates nucleotide signaling, nitrogen metabolism, and chaperone hubs. (**a**) EMSA of DasR binding to its target gene and the transcription levels of the known genes critical for nucleotide metabolism in *M. tuberculosis* H37Ra. (**b**) EMSA of DasR binding to its target gene and the transcription levels of the known genes critical for nitrogen metabolism in *M. tuberculosis* H37Ra. (**c**) EMSA of DasR binding to its target gene and the transcription levels of the known genes critical for chaperone binding in *M. tuberculosis* H37Ra. (**d**) EMSA of DasR binding to its target gene and the transcription levels of the known genes critical for acid stress response in *M. tuberculosis* H37Ra. EMSAs with a 200-fold excess of the unlabeled specific probe (S) or nonspecific competitor DNA (sperm DNA) (N) were used as controls. The *M. tuberculosis* H37Ra WT and Δ*dasR* strains were grown until the late exponential growth phase (OD_600_ = 1.0) in liquid MB7H9 medium. The fold change is the expression level compared with that of the WT strain. Data are presented as the means ± SDs for *n* = 3 biological replicates. ****, *P* < 0.0001; ***, *P* < 0.001; **, *P* < 0.01; *, *P* < 0.05; ns, *P* > 0.05; Student’s two-tailed *t*-test.

To directly examine the effects of disrupting DasR-mediated regulation, the expression of nucleotide metabolism-related genes, including *ppx1*, *ppx2*, *relA*, *cya*, *cpdA*, and *disA*, was subsequently verified by EMSAs and real-time RT-PCR monitoring of transcription. As shown in Fig. 3a, the *M. tuberculosis* Δ*dasR* strain exhibited graded transcriptional downregulation of the target genes. Furthermore, the transcription of nitrogen metabolism-associated genes (e.g., *narG*, *nirB*, *gltB*, and *glnA*) was strongly and positively correlated with *dasR* expression, indicating that DasR directly and positively regulates cellular nitrogen metabolism in *M. tuberculosis* (Fig. 3b). Considering the critical role of nitrogen assimilation in *M. tuberculosis* survival within macrophages (26), DasR-mediated transcriptional control of nitrogen assimilation genes may potentiate mycobacterial adaptation to glutamine-limited environments. DasR transcriptionally activates the chaperone HtpG (Fig. 3c), suggesting that it may regulate protein stability by influencing chaperone binding pathways. In addition, the transcript levels of acid stress genes were directly coordinated with *dasR* expression, implicating DasR in the direct control of acid adaptation (Fig. 3d). Based on the observation that *DasR* knockout *M. tuberculosis* strains exhibit compromised survival under oxidative stress and impaired virulence in guinea pig infection models (27), combined with our experimental data, we propose that *dasR* acts as a central transcriptional hub that integrates second-messenger signaling, nitrogen metabolism, and chaperone responses within the acid–stress adaptation network.

### pH-responsive amplification of DasR transactivation by c-di-AMP

**Fig 4:**
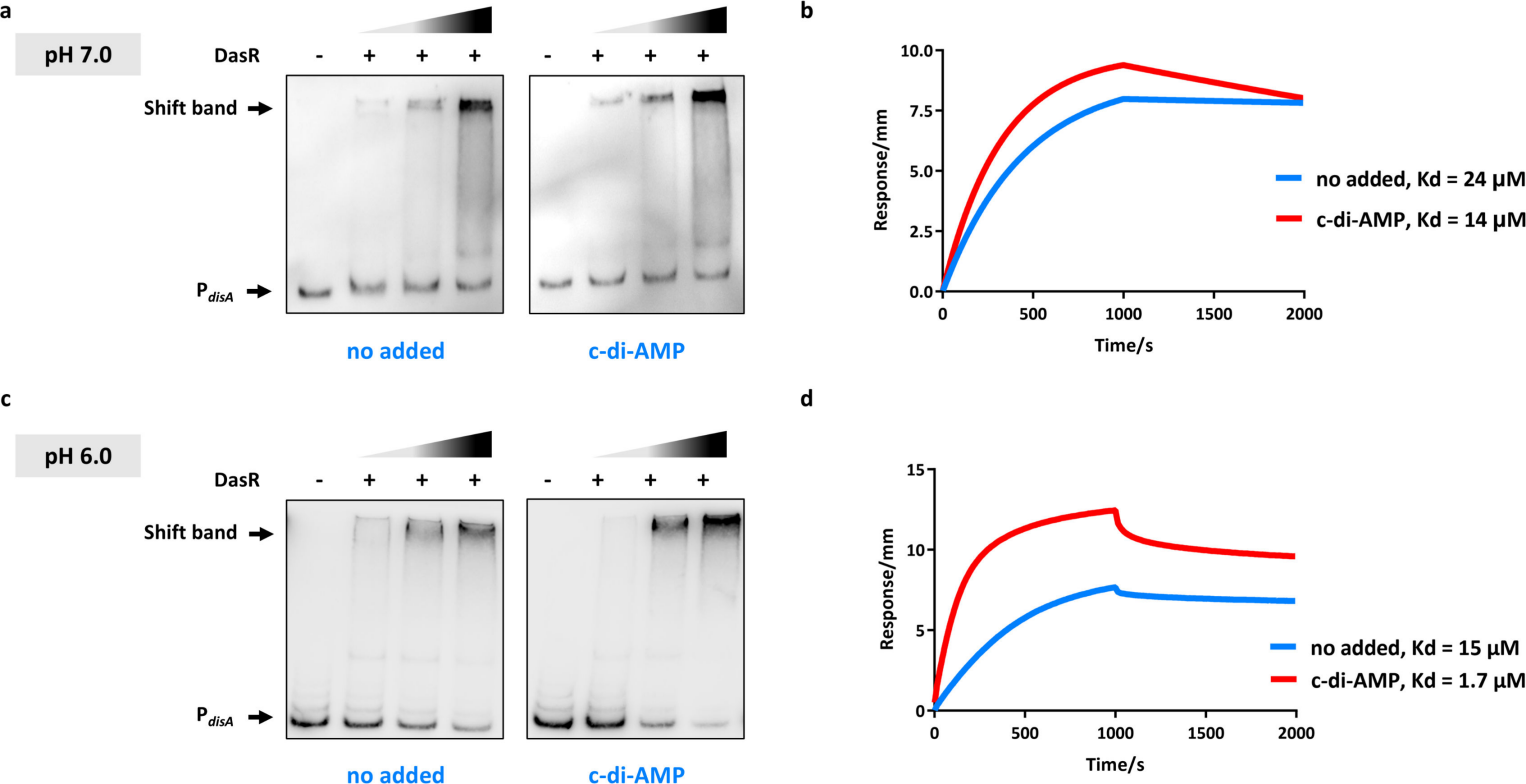
Acidic pH and c-di-AMP synergistically potentiate DasR-DNA binding. (**a**) EMSA of DasR binding to its target gene *disA* promoter. Purified His-DasR and DNA fragments were incubated with 300 μM c-di-AMP (right) or without c-di-AMP (left) at pH 7.0. Representative images of two independent experiments with similar results are shown. (**b**) BLI assay of purified His-DasR and DNA fragments with 300 μM c-di-AMP (pH 7.0). (**c**) EMSA of DasR binding to its target gene *disA* promoter. Purified His-DasR and DNA fragments were incubated with 300 μM c-di-AMP (right) or without c-di-AMP (left) at pH 6.0. Representative images of two independent experiments with similar results are shown. (**d**) BLI assay of purified His-DasR and DNA fragments with 300 μM c-di-AMP (pH 6.0).

Intriguingly, ChIP-seq analysis identified *disA* as a direct DasR target gene, and acidic stress triggered DasR-dependent *disA* transcriptional activation. These findings suggest that elevated c-di-AMP (synthesized by DisA) may exert feedback regulation on the DasR-mediated regulon. To test this hypothesis, we subjected the promoter of the *disA* gene to EMSA with DasR in the absence or presence of c-di-AMP at different pH values. In the absence of c-di-AMP, DasR formed a relatively stable complex with DNA fragments at relatively low protein concentrations, as evidenced by a clear mobility shift (Fig. 4a). Additionally, a biolayer interferometry (BLI) assay confirmed that DasR and DNA had a binding affinity of 24 μM in the absence of c-di-AMP, whereas the Kd was 14 μM after preincubation with c-di-AMP (Fig. 4b). Further analysis confirmed the acid-potentiated increase in DasR-DNA binding, with c-di-AMP increasing the binding affinity more than eightfold (Kd = 1.7 µM versus 15 μM at pH 6.0; Fig. 4c and d). These results indicated that the c-di-AMP-mediated, pH-responsive potentiation of DasR transactivation may be important for mycobacterial adaptation to acidic stress.

### Acid–stress remodeling of DasR function via chaperone coupling

**Fig 5:**
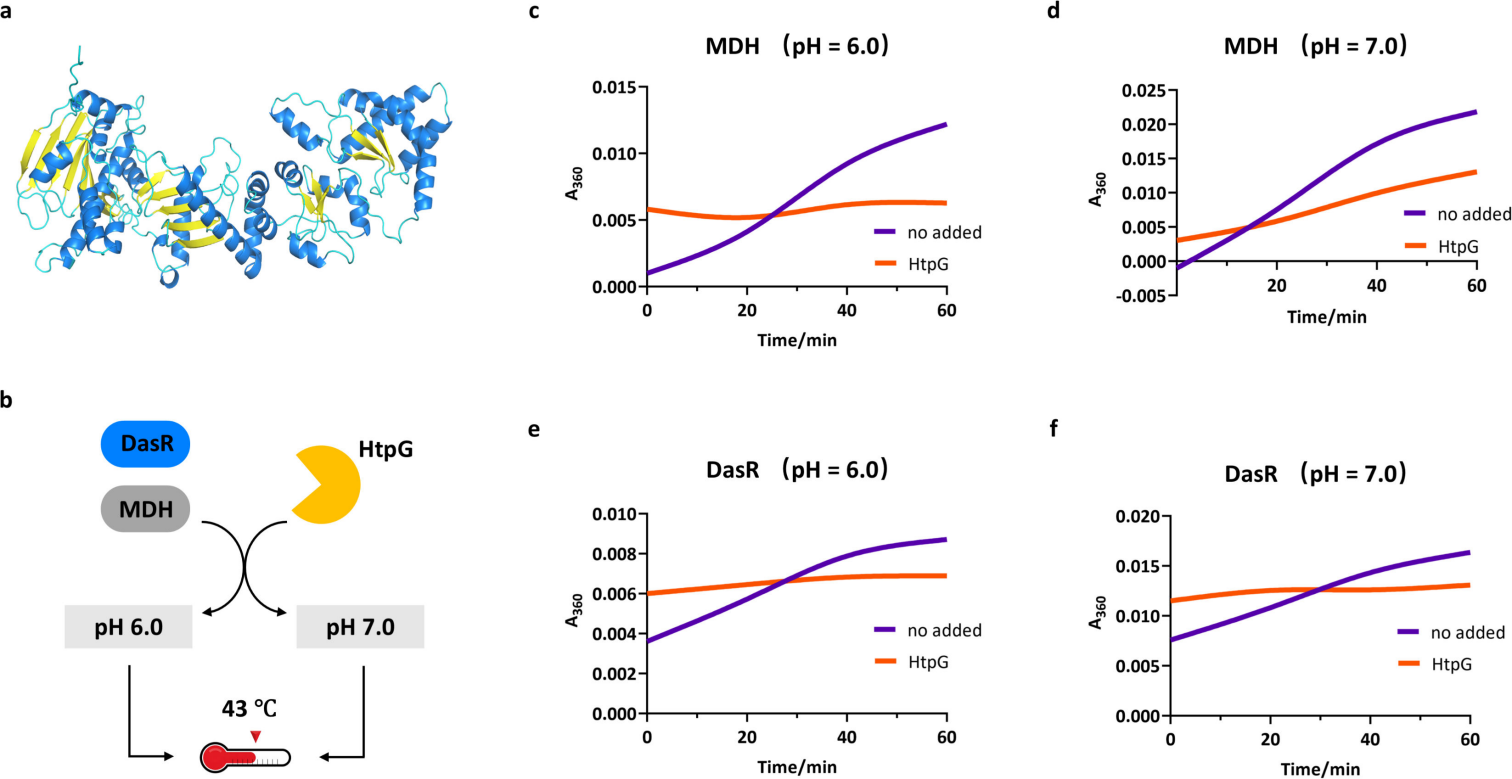
HtpG stabilizes the tertiary structure of DasR under acidic stress. (**a**) Model of *M. tuberculosis* HtpG obtained from AlphaFold2. (**b**) Schematic representation of the protein thermal stability workflow. Aggregation of MDH (heat-denatured at 43°C) monitored by light scattering (360 nm) in the presence or absence of HtpG at pH 6.0 (**c**) and at pH 7.0 (**d**). Aggregation of DasR (heat-denatured at 43°C) monitored by light scattering (360 nm) in the presence or absence of HtpG at pH 6.0 (**e**) and at pH 7.0 (**f**).

Molecular chaperones maintain proteostasis by facilitating protein folding, preventing aggregation, and promoting refolding/degradation under stress conditions. In addition to c-di-AMP-mediated DasR transactivation, chaperone binding by HtpG could provide another mechanism of DasR function. Early studies revealed that HtpG (Fig. 5a), a member of the Hsp90 chaperone family, plays key roles in protecting *M. tuberculosis* from stress and activating an immune response (28). Using a thermal aggregation assay (Fig. 5b), we first evaluated the effect of HtpG on the aggregation of thermally denatured malate dehydrogenase (MDH) (29, 30) under acidic versus neutral conditions (pH 6.0 versus pH 7.0). The results showed that HtpG significantly reduced MDH aggregation in a pH-dependent manner (Fig. 5c and d). Building on these findings, we demonstrated that HtpG also inhibits the thermoaggregation of DasR at 43°C, with markedly stronger protection observed at pH 6.0 than at pH 7.0. This suppression of DasR aggregation by HtpG was further confirmed by light scattering assays (360 nm), which revealed consistently less aggregation at pH 6.0 (Fig. 5e) than at pH 7.0 (Fig. 5f).

## DISCUSSION

Our study identifies DasR as a c-di-AMP receptor in *M. tuberculosis* that exhibits significantly enhanced binding affinity under acidic conditions. This pH sensitivity is correlated with the acidic phagosomal microenvironment encountered during host infection, positioning the c-di-AMP–DasR axis as a dedicated pH-sensing module. Notably, although c-di-AMP receptors such as CabP (*Streptococcus pneumoniae*) or DarR (*Mycobacterium smegmatis*) regulate osmoadaptation (31, 32), DasR represents the first actinobacterial transcription factor demonstrating pH-dependent c-di-AMP binding—a mechanism likely conserved across Actinobacteria given the ubiquitous presence of its c-di-AMP-binding domain in genera such as *Streptomyces* and *Corynebacterium* (19).

In addition to its role in receptor‒ligand interactions, a genome-wide ChIP-seq analysis revealed that DasR directly coordinates nucleotide second-messenger homeostasis. Specifically, DasR targets genes that control (p)ppGpp (*relA*), cAMP (*cya*), and c-di-AMP (*disA*) metabolism, enabling synchronized stress signaling under acid stress. This multiplexed control contrasts with that of canonical single-messenger regulators (e.g., CRP-cAMP in *Escherichia coli* or *Salmonella enterica*) (33, 34), reflecting the sophisticated adaptation demands of persistent mycobacteria. Critically, we observed that c-di-AMP allosterically potentiates DasR-DNA binding—an effect markedly amplified at low pH—establishing a feed-forward loop in which DasR upregulates *disA* to increase c-di-AMP pools. This subsequently increases the transcriptional output of DasR. Such positive feedback diverges from the negative DisA–phenethylamine feedback loop in *Proteus mirabilis* (35), suggesting lineage-specific optimization for rapid environmental response.

**Fig 6:**
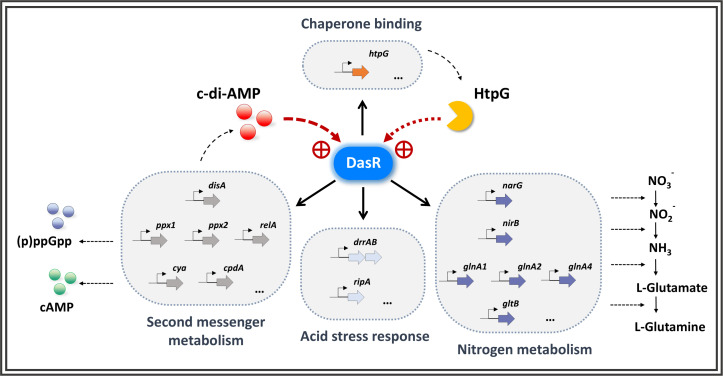
Working model for the c-di-AMP–DasR signaling axis in *M. tuberculosis*. Model depicting DasR as a pH-sensitive receptor that binds c-di-AMP with strongly enhanced affinity under acidic conditions. Ligand-bound DasR directly targets the promoters of nucleotide metabolism genes and chaperones, enabling the coordinated control of acid adaptation. The system forms a self-reinforcing circuit: c-di-AMP allosterically promotes DasR-DNA binding, DasR upregulates c-di-AMP synthesis, and the chaperone HtpG stabilizes DasR under stress. The arrows indicate direct transcriptional regulation.

DasR directly activates the molecular chaperone HtpG (a bacterial Hsp90 homolog). Under acidic conditions, HtpG stabilizes the structure of DasR and synergizes with c-di-AMP to increase its transcriptional activity. Although this chaperone–transcription factor coupling resembles that in eukaryotic Hsp90-mediated steroid receptor stabilization (36), such mechanisms remain less characterized in bacteria. While the role of HtpG in heat shock proteostasis is well established (30), its specific function in the protection of transcriptional regulators during acidic stress represents a key finding. Physiologically, *dasR-*knockout *M. tuberculosis* strains (where DasR, encoded by MRA_0802, is 100% identical to Rv792c) exhibit compromised survival under oxidative stress and impaired virulence in guinea pig infection models (27). Collectively, these components establish a dual-reinforcement system combining ligand-enhanced DNA binding and chaperone-mediated structural stabilization to ensure signaling fidelity in acidic environments (Fig. 6).

In a broader context, our work extends the functional repertoire of c-di-AMP beyond osmoregulation and cell wall maintenance, revealing its role as a pH-responsive transcriptional orchestrator. Compared with its canonical function in potassium uptake regulation (37), DasR-mediated control of multiple messengers implies a higher-order regulatory tier in Actinobacteria. Therapeutically, disrupting the c-di-AMP–DasR interaction could attenuate acid tolerance in persistent tuberculosis, potentially offering advantages over proton pump inhibition by targeting pathogen-specific signaling. Future studies should prioritize determining the high-resolution structure of DasR in its apo and c-di-AMP bound states. This would reveal the conformational changes upon ligand binding and pinpoint druggable pockets. Structural-based virtual screening and fragment-based drug design could subsequently be employed to identify lead compounds that lock DasR in an inactive conformation, thereby ablating its regulon and attenuating virulence.

## MATERIALS AND METHODS

### Bacterial strains and culture conditions

The strains and plasmids used in the experiments are listed in Table S1. *M. tuberculosis* H37Ra strains were routinely maintained in Middlebrook 7H9 broth medium (MB7H9) (Difco). MB7H9 medium was supplemented with 10% albumin-dextrose-catalase (ADC; Difco), Tween-80 (0.05%), and glycerol (0.2%) (MB7H9 + ADC). The *E. coli* strains were cultured in LB medium (10 g of NaCl, 5.0 g of yeast extract, and 10.0 g of tryptone in 1 L of ddH_2_O) at 37°C with shaking at 220 rpm. The corresponding culture conditions used were based on the experimental needs.

### Overproduction and purification of proteins *in vitro*

The *dasR* gene was amplified from the genomic DNA of *M. tuberculosis* H37Ra by PCR using the primers listed in Table S2. After restriction digestion with EcoRI and HindIII, the gene coding for *dasR* (MRA_0802) was cloned and inserted into pET28a. The protein was expressed by the *E. coli* BL21(DE3) strain and purified as previously described. The His-tagged protein was purified by Ni-NTA Superflow columns (Qiagen) and eluted with 250 mM imidazole (in 50 mM NaH_2_PO_4_, 300 mM NaCl, pH 8.0). The protein concentration was determined using a BCA Protein Assay kit (Tiangen) with BSA as the standard (38).

### Construction of the *M. tuberculosis* H37Ra Δ*dasR* strain

The *dasR* (MRA_0802) gene was replaced with a hygromycin resistance cassette (*hyg*) and a *sacB* counterselectable marker using the allelic exchange method (39). The upstream and downstream homology arms of the MRA_0802 gene were amplified by PCR from the *M. tuberculosis* H37Ra genome using the specific primers *dasR*-ko-uF/uR and *dasR*-ko-dF/dR. The amplified upstream and downstream homology arms, together with the hygromycin resistance-*sacB* gene cassette, were cloned and inserted into the suicidal vector p0004S, and the resulting recombinant cosmid p0802S containing AES was used to construct the bacteriophage. The cosmid was inserted into the temperature-sensitive shuttle plasmid, phAE159, to construct specialized transducing phages. The recombinant phAE159 was subsequently electroporated into *M. smegmatis* mc^2^155 competent cells to obtain high-titer phages. The *M. tuberculosis* H37Ra strain was infected with phages and then cultured in solid medium containing *hyg* resistance at 37°C for 4 weeks. Monoclones were screened and verified by PCR. Complemented strain was constructed by electroporation of *dasR* cloned in pMV261 vector. Complementation was then confirmed by PCR. The primers used are listed in Table S2.

### EMSA

DNA fragments spanning the approximately 150 bp promoter region from upstream of DasR target genes were amplified by PCR using the primers listed in Table S2. The PCR products were modified with biotin with a primer (5′-biotin-AGCCAGTGGCGATAAG-3′) (40). The biotin-labeled PCR products were subsequently purified with a PCR purification kit (Shanghai TransGen Biotech, China). EMSAs were carried out as previously described with a chemiluminescent EMSA kit (Beyotime Biotechnology, China). The binding reaction contained 10 mM Tris–HCl, 25 mM MgCl_2_, 50 mM NaCl, 1 mM DTT, 1 mM EDTA, 0.01% Nonidet P40, 50 g/mL^−1^ poly[d(I-C)], and 10% glycerol. After binding, the samples were separated on a nondenaturing PAGE gel in an ice bath of 0.5× Tris–borate–EDTA at 100 V.

### BLI assay

The biosensor streptavidin (SA) purchased from ForteBio was used in this work. The loading buffer (pH 7.0 or 6.0) contained 10 mM HEPES, 2 mM MgCl_2_, 0.1 mM EDTA, and 200 mM KCl, and the running buffer contained an extra 10 g/mL BSA and 0.02% Tween-20. The biotin-labeled DNA probe used was the same as that used for EMSAs. The DNA probe was stored in loading buffer, and His-tagged DasR was stored in running buffer during the BLI assay with SA sensors. Samples were then detected within the OptiPlate-96Black Opaque (PerkinElmer) (41).

### ITC experiment

Purified DasR protein was titrated with c-di-AMP (Medmol, China, CAS:54447-84-6) in ITC200. All the samples were prepared in PBS buffer (pH 7.0 or 6.0). Typically, the titrant concentration in the syringe ranged from 200 μM to 1,000 μM, and the titrant concentration in the reaction cell ranged from 10 μM to 50 μM. Titration was conducted at 25°C using a multiple injection method with 150 s intervals. The obtained data were integrated, corrected, and analyzed using MicroCal PEAQ-ITC analysis software with a single-site binding model.

### c-di-AMP quantification

Bacteria were harvested by centrifugation (8,000 × *g*, 30 min), washed, and lyophilized for dry weight measurement to standardize subsequent analyses. The pellet was resuspended in 15–20 mL of ice-cold extraction buffer (ether/methanol/water, 40:40:20, vol/vol; LC-grade, VWR) and rapidly frozen in liquid nitrogen for 10 min. After thawing, the samples were heated at 95°C for 10 min and then incubated at 4°C for 30 min. Cell lysis was performed using an ultrasonic disruptor for 15 min. The supernatant was collected and stored at 4°C. The remaining pellet was re-extracted with buffer, incubated on ice for 30 min, and heated again. Following centrifugation, the supernatant was combined with the previous fraction. The pooled supernatant was lyophilized and reconstituted in 500 μL of extraction buffer for HPLC analysis using an RPC-18 column (250 × 4.5 mm; Kromasil). c-di-AMP concentrations were normalized to dry cell weight, and intracellular levels were calculated based on an estimated cellular volume of 7.14 mL/g dry weight, adapted from *E. coli* (42).

### ChIP-seq and data analysis

The *M. tuberculosis* H37Ra WT strain was grown for the appropriate length of time. Formaldehyde was added to cultures at a final concentration of 1% (vol/vol), and incubation was continued for 30 min. Glycine was then added to a final concentration of 125 mM to stop the cross-linking. The samples were left at room temperature for 10 min and washed twice in the precooled TBS buffer containing 20 mM Tris–HCl and 150 mM NaCl. The samples were then subjected to the ChIP-seq performed by E-GENE Tech Co., Ltd. (Shenzhen) using an anti-DasR polyclonal antibody. The specificity of the anti-DasR antibody was confirmed by Western blot analysis, which revealed a highly specific signal at the predicted size with minimal cross-reactivity (Fig. S4). For the data analysis, fastp software (v0.20.0) was used to trim the adaptors and remove low-quality reads to obtain high-quality clean reads. Clean reads were aligned to the reference genome using Bowtie2 software (v2.2.4). MACS2 software (v2.2.7.1) was used for peak calling. Bedtools software (v2.30.0) was used for peak annotation based on GTF annotation files. Homer (v4.11) software was used to identify the motifs. MAnorm2 (v1.2.0) software was used to identify differentially enriched regions. The enriched peaks were visualized with IGV (v2.14.1) software.

### Thermal aggregation assay

To investigate thermally denatured protein aggregation, 0.5 μM protein was introduced into 2 mM DTT and 40 mM HEPES–KOH buffer and prewarmed to 43°C, at either pH 7.0 or 6.0. This was carried out with or without 0.25 μM HtpG, while stirring continuously. The aggregation of protein was monitored by measuring light scattering at 360 nm. A lumina fluorescence spectrometer equipped with a Peltier temperature controller was utilized for this purpose (43).

### Real-time RT-PCR

Bacteria grown at the indicated time points were harvested by centrifugation (12,000 × *g*, 4°C for 5 min), and an RNA prepPureCell/Bacteria kit (Tiangen Biotechnology, China) was used for RNA extraction. The RNA concentration was measured by a microplate reader (Bio Tek, USA). Total RNA (1 μg) was reverse transcribed using the PrimeScript RT Reagent kit and gDNA Eraser (Takara). PCR was performed by using a CFX96 real-time system (Bio-Rad, USA). The following thermal cycling conditions were used: 95°C for 5 min, 95°C for 15 s, 58°C for 15 s, and 72°C for 30 s for 40 cycles. The primers used are listed in Table S2, and *sigA* was used as an internal control (19).

### Molecular docking analysis

The 3D structure of DasR predicted by AlphaFold and the c-di-AMP structure from PubChem were prepared for docking using AutoDockTools (ADT). Preprocessing involved removing water molecules, adding hydrogens, and calculating charges. Docking was executed with AutoDock Vina, with a grid box defined to cover the predicted active site of DasR. The top-ranked conformation selected on the basis of the lowest binding energy was analyzed using PyMOL to identify key interactions and generate structural representations.

### Statistical analysis

Data from at least three independent experiments are shown as the mean ± SD. Statistical significance was determined using Student’s *t*-test (two groups) or one-way ANOVA (multiple groups), with *P* < 0.05 considered significant.

## Data Availability

Data representations can be found in the article and the supplemental material. The ChIP-seq data used in this study are available in the GEO database (https://www.ncbi.nlm.nih.gov/geo/query/acc.cgi?acc=GSE306352). The DasR targets identified by ChIP-seq can be found in Table S3.

## SUPPLEMENTAL MATERIAL

Supplemental material mbio.03708-25-s0001.docx Supplemental figures and tables.

Table S3 mbio.03708-25-s0002.xls The DasR targets identified by ChIP-seq.

ASM does not own the copyrights to Supplemental Material that may be linked to, or accessed through, an article. The authors have granted ASM a non-exclusive, world-wide license to publish the Supplemental Material files. Please contact the corresponding author directly for reuse.
