# Supplementary material for: c-di-AMP–DasR signaling axis mediates mycobacterial acid resistance
Source: mBio. 2026 Feb 6;17(3):e03708-25. doi: 10.1128/mbio.03708-25 (PMC12977524; doi:10.1128/mbio.03708-25)
Supplement: Supplemental material — Supplemental figures and tables. [file mbio.03708-25-s0001.docx]

Supplementary Information:

**c-di-AMP–DasR signaling axis mediates Mycobacterial acid resistance**

Yu Fu^1^, Xue-Qin Xie^1^, Zhan-Hui Xu^1^, Yi-Fan Liang^1^, Shi-Qi Yang^1^, Bang-Ce Ye^1^, Di You^1*^

^1^Laboratory of Biosystems and Microanalysis, State Key Laboratory of Bioreactor Engineering, East China University of Science and Technology, Shanghai 200237, China;

^*^ Corresponding author

**Corresponding authors**

Di You

Associate Professor, Lab of Biosystems and Microanalysis,

State Key Laboratory of Bioreactor Engineering,

East China University of Science and Technology, Shanghai 200237, China

Tel/Fax: 0086-21-64253832

Email: 030111115@mail.ecust.edu.cn

**
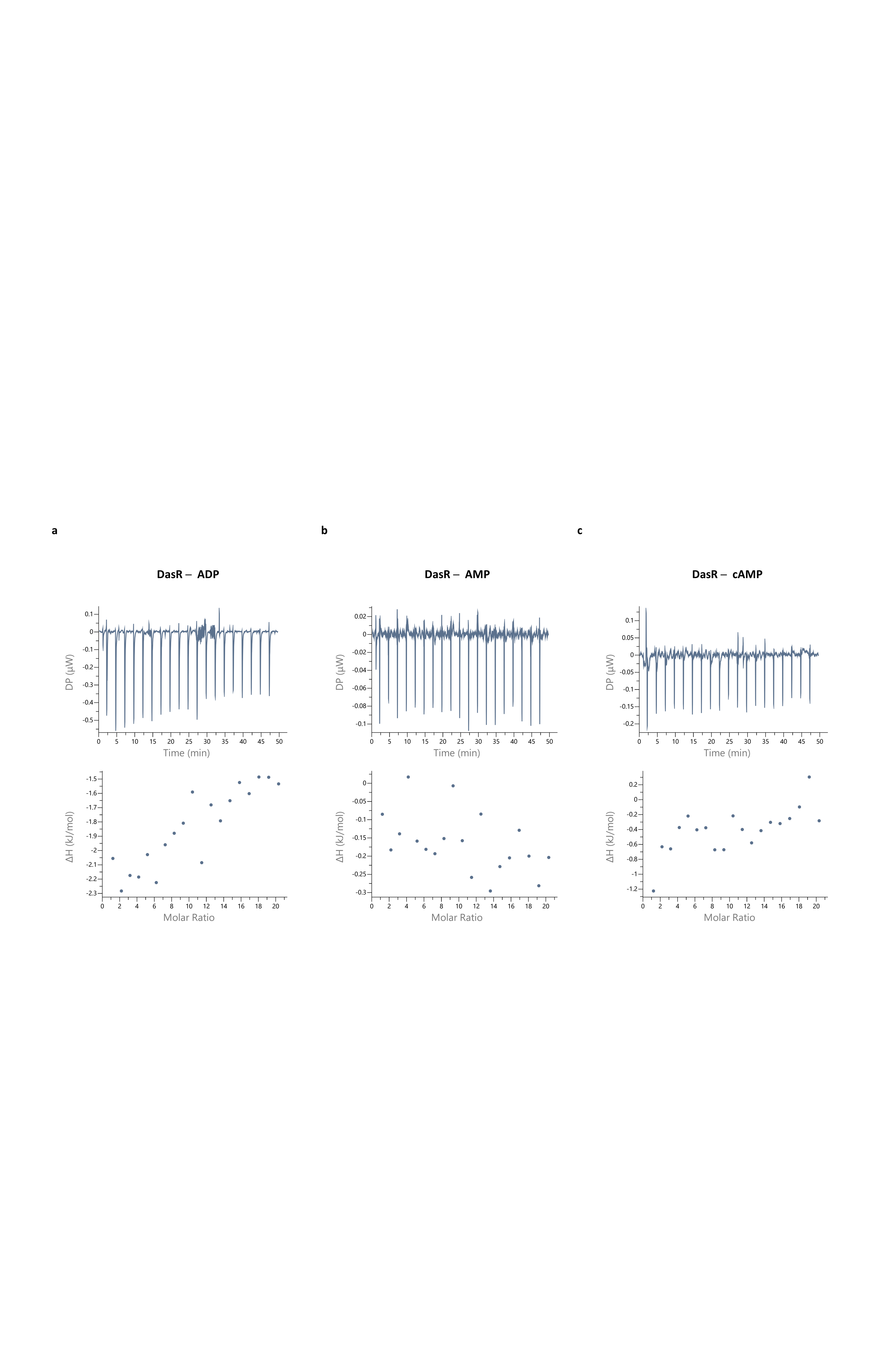
**

**Supplementary Figure 1**. Characterization of the interactions of nucleotides with DasR using ITC. Titration of nucleotides (1 mM) into DasR (10 μM). ITC measurements of DasR titrated with ADP (**a**), AMP (**b**), and cAMP (**c**).

**
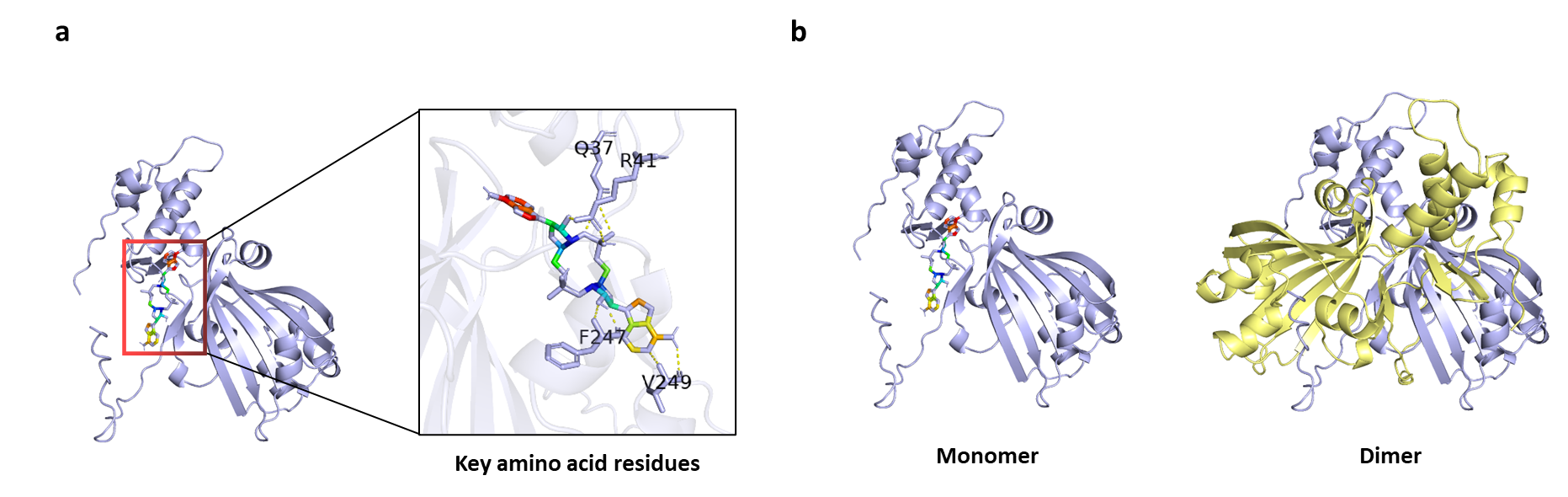
**

**Supplementary Figure 2**. Predicted interaction between c-di-AMP and DasR. (a) The top-ranking docking pose of c-di-AMP bound to the effector domain of DasR (binding energy: -6.4 kcal/mol). Key interacting residues are indicated. (b) The AlphaFold2-predicted dimeric architecture of DasR.


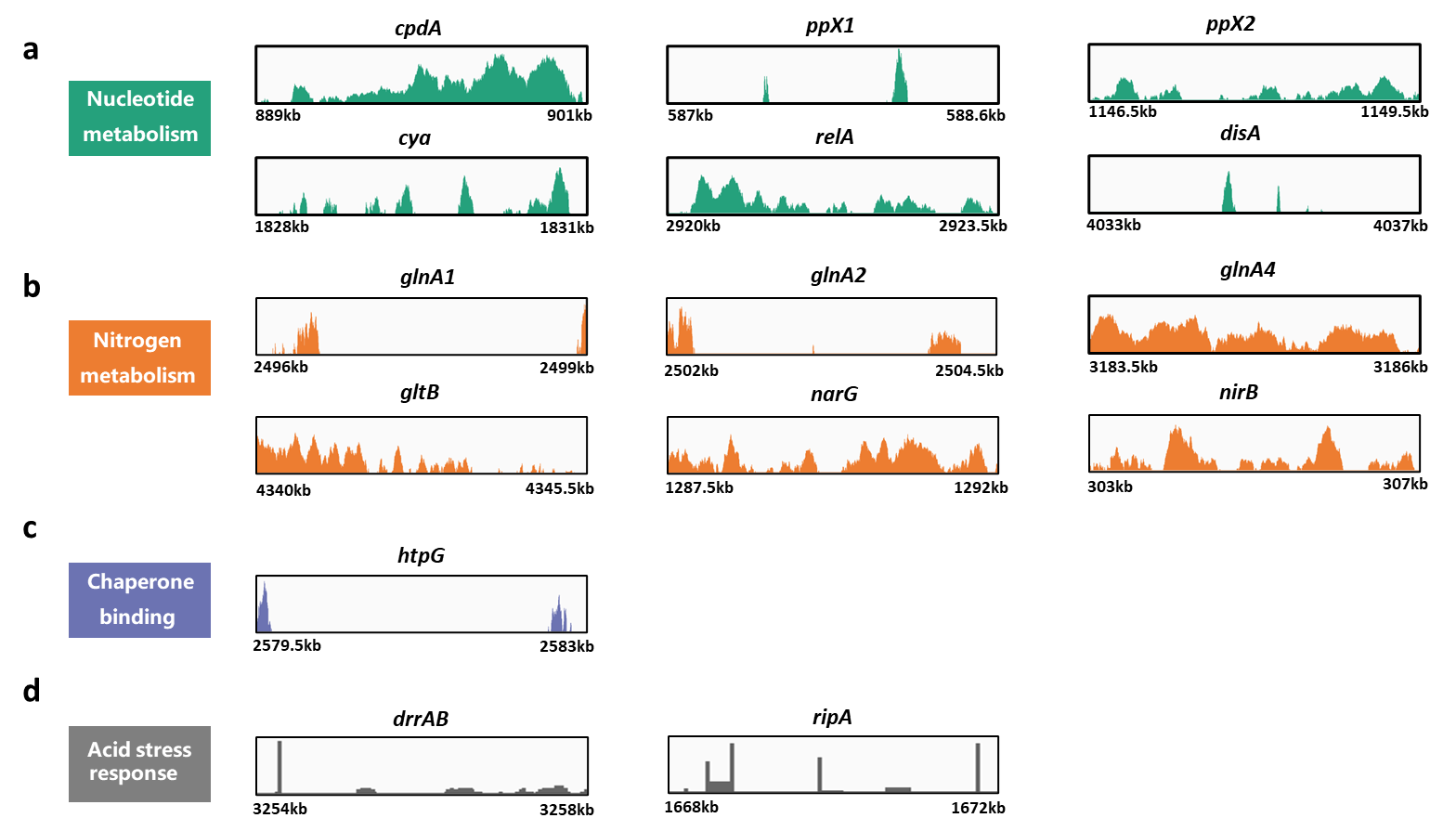


**Supplementary Figure 3.** Genome browser views of DasR binding at selected genomic loci. (a-d) IGV snapshots of the ChIP-seq data confirming DasR occupancy at the promoters of key genes, corresponding to the peak calling results in Fig. 2. Representative genes involved in (a) nucleotide metabolism, (b) nitrogen metabolism, (c) chaperone function, and (d) acid stress response are shown.


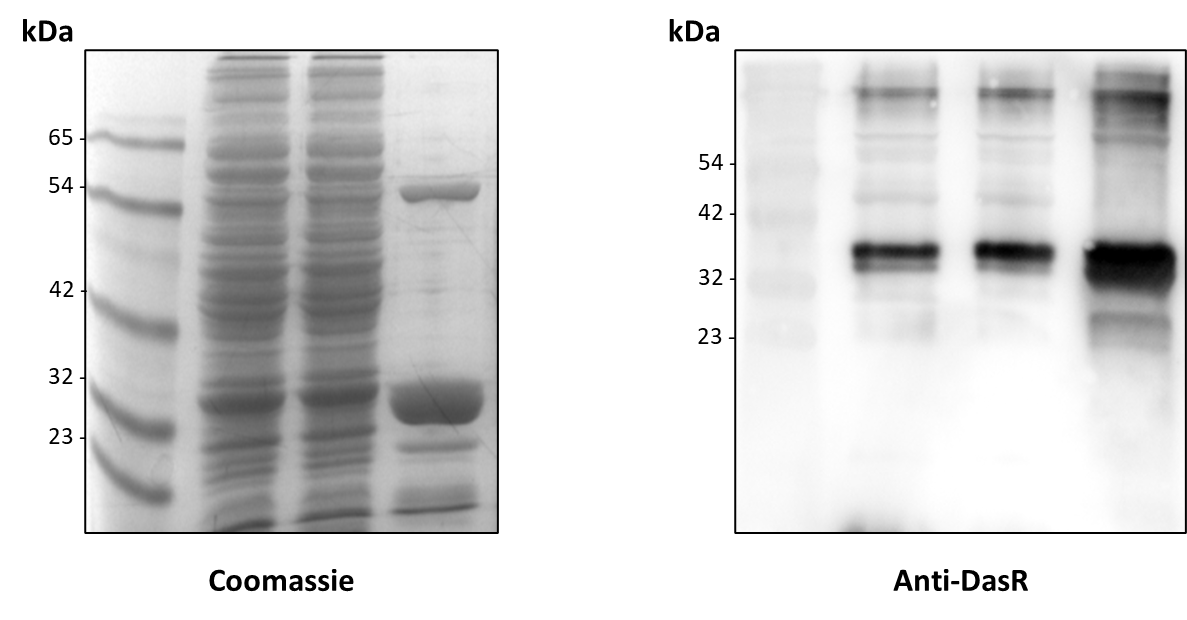


**Supplementary Figure 4**. Validation of the anti-DasR antibody by Western blot. Lanes from left to right: protein marker, total lysate, culture supernatant, and purified recombinant DasR protein.

| **Strains or plasmids** | **Reference or source** |
| --- | --- |
| Strains |  |
| *M.tuberculosis* H37Ra | This study |
| *M.tuberculosis* H37Ra Δ*dasR* | This study |
| *E. coli* BL21 (DE3) | TransGen |
| BL21(DE3)/pET-*dasR* | This study |
| *M. smegmatis* mc^2^155 | This study |
| Plasmids |  |
| pET-28a-*dasR* | This study |
| pET-28a(+) | Tsingke |
| p0004S | (39) |
| p0802S | This study |
| phAE159 | (39) |
| Δ*dasR*::*dasR* | This study |

**Supplementary Table 1.** Strains and plasmid

**Supplementary Table 2. P**rimers used in this work

| **Primers for overproduction of proteins** | |
| --- | --- |
| Primer name | Sequence (5’ to 3’) |
| pET-*dasR*-F | TGCCGCGCGGCAGCCATATGATGACATCTGTCAAGCTGGAC |
| pET-*dasR*-R | TGTTAGCAGCCGGATCTCATCATGCGAAATCTCGTTTCTCGAT |
| **Primers used to validate plasmid construction** | |
| Primer name | Sequence (5’ to 3’) |
| T7-F | TAATACGACTCACTATAGGGGAATTGTGAGCG |
| T7-R | CAAAAAACCCCTCAAGACCCGTTTAGAGG |
| **Primers for knocking out and verifying *dasR*** | |
| *dasR*-ko-uF | CACGTTCAGGCCCTGGGCATGCT |
| *dasR*-ko-uR | ACGCCAAAGACGACCCGCACTTCGGAC |
| *dasR*-ko-dF | AAGACGTATTAAAACGTCTTATACTCACCACG |
| *dasR*-ko-dR | TGAGACTTCTTGAATCCTACCGAGATTCGTTTTA |
| *hyg*-F | CGGGTCGTCTTTGGCGTatgaacatcaaaaagtttgcaaaa |
| *hyg*-R | TAAGACGTTTTAATACGTCTTttcctttgccctcggacgagtgct |
| **Primers for supplementing DasR** | |
| Δ*dasR*::*dasR* -F: | GCCCCGGCTCGGGGATGG |
| Δ*dasR*::*dasR*-R: | CCATCACAGAGGTGTTGTTGAGCTGT |
| **Primers for EMSA with biotin labeling** | |
| Primer name | Sequence (5’ to 3’) |
| Universal primer | Biotin-AGCCAGTGGCGATAAG |
| *ppx*1-F | AGCCAGTGGCGATAAGCGCGGGATTCCGCCATAGCCCA |
| *ppx*1-R | AGCCAGTGGCGATAAGCCAGCAGATGGACCGTGTTGCTACC |
| *ppx*2-F | AGCCAGTGGCGATAAGCGCCGAGATGGAACAACTGGCTGC |
| *ppx*2-R | AGCCAGTGGCGATAAGCAAAGCCGAGCCGTTCGCGG |
| *relA*-F | AGCCAGTGGCGATAAGGGTGGAACTTGCGGGGTTGAGCG |
| *relA*-R | AGCCAGTGGCGATAAGCGCTTGCGCCGTGAGCTGGTC |
| *cpdA*-F | AGCCAGTGGCGATAAGACTCGACTGTGATCGCGGGGTC |
| *cpdA*-R | AGCCAGTGGCGATAAGGCACCTGAGGTGCCAACCCTCT |
| *cya*-F | AGCCAGTGGCGATAAGACGGACTCAAAACCCGTCCAG |
| *cya*-R | AGCCAGTGGCGATAAGGTTCGTGGCGGCAAGAAAATGC |
| *disA-F* | AGCCAGTGGCGATAAGTGCGGTTACGGCTGCCCTGA |
| *disA-R* | AGCCAGTGGCGATAAGCGAATCGGTTCTTGACCCCG |
| *nirB*-F | AGCCAGTGGCGATAAGCTGAGTCGACCACGCTTAAGTTCC |
| *nirB*-R | AGCCAGTGGCGATAAGGGGGCAAATTACCGAAAAATCGCC |
| *narG*-F | AGCCAGTGGCGATAAGCCGCCGCAGATGTCCACCGTC |
| *narG*-R | AGCCAGTGGCGATAAGCATCGTAATCGCTACGGGCATGCAA |
| *gltB*-F | AGCCAGTGGCGATAAGTCGAATCCGCAAAGGCGGCCG |
| *gltB*-R | AGCCAGTGGCGATAAGTGCATGTCCACAACCATGGCAAC |
| *glnA*1-F | AGCCAGTGGCGATAAGGGATAGTCGGGCGGCGATTTCG |
| *glnA*1-R | AGCCAGTGGCGATAAGCCACTGACGCCGGGTCTCCC |
| *glnA*2-F | AGCCAGTGGCGATAAGAACTCACCGCGCACTCCCCTT |
| *glnA*2-R | AGCCAGTGGCGATAAGCGCGTTCTTCCAGGGTACGAAGAA |
| *glnA*4-F | AGCCAGTGGCGATAAGGCCGGAACGCCTCCCGCTCTTC |
| *glnA*4-R | AGCCAGTGGCGATAAGTCCCGAGAGTTCTGGCACGGCTTC |
| *htpG*-F | AGCCAGTGGCGATAAGCGGATCGCAGCTCGCTAAATTCGA |
| *htpG*-R | AGCCAGTGGCGATAAGAGATCAACTCCCGCAGAAACGCGT |
| *drrA/B-F* | AGCCAGTGGCGATAAGCTGCTGCGCTACGTTTACGCCCC |
| *drrA/B-R* | AGCCAGTGGCGATAAGGATACACCCGAAGTTCGATGGCGTG |
| *ripA-F* | AGCCAGTGGCGATAAGTCCGGTGCAGTCCGCGCA |
| *ripA-R* | AGCCAGTGGCGATAAGAACTGCCCCCGGTACGTGC |
| **Primers for RT-PCR** | |
| Primer name | Sequence (5’ to 3’) |
| RT*ppx*1-F | AGGACCCTAACGGCAAATGG |
| RT*ppx*1-R | ATGCTCGCCTCTGCCACCAG |
| RT*ppx*2-F | GGCTGGCTGGAACGATGACC |
| RT*ppx*2-R | CACCCGCGAAAGATGAATGG |
| RT*relA*-F | GCCGACCTGTCGATCTTGC |
| RT*relA*-R | CGGGTGGGTGATGTAGGGAT |
| RT*nirB*-F | CGATTGCCGACGGTTGTGG |
| RT*nirB*-R | ACAGCGCCTTGGACTGCTCC |
| RT*narG*-F | GGAGCCCGTTCTATCCACAA |
| RT*narG*-R | CGGCGACGTAATCACCCAC |
| RT*gltB*-F | GCGGCAGGGTTGTCATTCT |
| RT*gltB*-R | CCGTCGGGATCGTAAACATAG |
| RT*sigA*-F | GACGAAGACCACGAAGAC |
| RT*sigA*-R | TCATCCCAGACGAAATCAC |
| RT*disA*-F | GCAAATCAATGCCACCC |
| RT*disA*-R | CTGCAACGTTCCGAACG |
| RT*cpdA*-F | CGATTCCGAGTGTTTTG |
| RT*cpdA*-R | TCGTCGAGTAGTGCAGG |
| RT*cya*-F | ACGGGCTGGAGAAAATC |
| RT*cya*-R | CCCCACACGTCGTAGAA |
| RT*dasR*-F | CTGGGTTTGGTTGAGGT |
| RT*dasR*-R | ATGTGGTTTCGGTGTCC |
| RT*glnA*1-F | CTTCAAACTTGCCAAGG |
| RT*glnA*1-R | GTCGTCAAACACGCTCT |
| RT*glnA*2-F | GGACCGACAGAAGGAAT |
| RT*glnA*2-R | CGACCGACTTGAGGAAA |
| RT*glnA*4-F | CGGCAACGCCTACCAAG |
| RT*glnA*4-R | CGTATCCTCTCCCAATC |
| RT*htpG*-F | AGGAAGAAGGGGGGGAA |
| RT*htpG*-R | GGGATGAACAGCAGGGC |
| RT*drrA/B*-F | TTGTTGACCACGCAGTATCTCG |
| RT*drrA/B*-R | CGGGGCACTATTTCGCAGA |
| RT*ripA*-F | CGGATTCCGCGAGTTTATGG |
| RT*ripA*-R | ACTGCGAACCCGAGTAGTGC |

**Supplementary Table 3.** The DasR targets identified by ChIP-seq.
